# High-quality genome assembly and annotation of the white-cheeked goby, *Rhinogobius duospilus* (Herre, 1935) (Gobiiformes: Oxudercidae)

**DOI:** 10.1093/g3journal/jkaf278

**Published:** 2025-11-18

**Authors:** Lixian Wu, Jiantao Hu, Shanshuo Zhang, Yi Qing Fam, Jianhong Xia, Chenhong Li

**Affiliations:** Shanghai Universities Key Laboratory of Marine Animal Taxonomy and Evolution, Shanghai Ocean University, Shanghai 201306, China; Engineering Research Center of Environmental DNA and Ecological Water Health Assessment, Shanghai Ocean University, Shanghai 201306, China; Shanghai Universities Key Laboratory of Marine Animal Taxonomy and Evolution, Shanghai Ocean University, Shanghai 201306, China; Engineering Research Center of Environmental DNA and Ecological Water Health Assessment, Shanghai Ocean University, Shanghai 201306, China; Shanghai Universities Key Laboratory of Marine Animal Taxonomy and Evolution, Shanghai Ocean University, Shanghai 201306, China; Engineering Research Center of Environmental DNA and Ecological Water Health Assessment, Shanghai Ocean University, Shanghai 201306, China; Shanghai Universities Key Laboratory of Marine Animal Taxonomy and Evolution, Shanghai Ocean University, Shanghai 201306, China; Engineering Research Center of Environmental DNA and Ecological Water Health Assessment, Shanghai Ocean University, Shanghai 201306, China; Shanghai Natural History Museum, Branch of the Shanghai Science & Technology Museum, 510 West Beijing Rd, Jing’an District, Shanghai 200041, China; Shanghai Universities Key Laboratory of Marine Animal Taxonomy and Evolution, Shanghai Ocean University, Shanghai 201306, China; Engineering Research Center of Environmental DNA and Ecological Water Health Assessment, Shanghai Ocean University, Shanghai 201306, China

**Keywords:** white-cheeked goby, *Rhinogobius duospilus*, Hi-C, chromosome-level genome assembly, creek fishes

## Abstract

The white-cheeked goby (*Rhinogobius duospilus*) is a small stream-dwelling fish endemic to southern China and Vietnam. With a sucker-like modified pelvic fin that helps it cling to substrate in fast-flowing water and vibrant breeding colors in males, *R. duospilus* is particularly appealing to aquarium enthusiasts. To investigate its distribution patterns, evolutionary history, and molecular adaptations to local environments, a high-quality genome assembly is critically needed. By employing PacBio HiFi sequencing combined with Hi-C-assisted assembly technology, we successfully obtained a chromosome-level genome assembly of *R. duospilus*. The final assembly yielded a genome size of 1,031.61 Mb with a scaffold N50 of 45.55 Mb. Approximately 991.84 Mb of genomic sequence was anchored onto 22 chromosome pairs. Benchmarking Universal Single-Copy Orthologs assessment indicated high genome completeness at 96.14%. Through gene prediction and functional annotation, we identified 24,418 protein-coding genes, with 23,660 (96.8%) successfully annotated. This work presents the first high-quality reference genome for *R. duospilus*, creating an essential genomic resource for investigating population differentiation and adaptive evolution through comparative genomics. Additionally, this dataset provides valuable support for taxonomy, evolution, and conservation genetics of genus *Rhinogobius*.

## Introduction

The white-cheeked goby (*Rhinogobius duospilus*) is a small benthic fish endemic to subtropical creeks in China and Vietnam (Pan et al. 1991). Ecologically, this species plays an essential role in the energy cycle of the freshwater ecosystem, feeding on small invertebrates while serving as prey for larger carnivorous species (Pan et al. 1991). Recently, *R. duospilus* has gained popularity among aquarium enthusiasts due to its specialized sucker-like pelvic fin, which allows it to cling to substrate in flowing water, as well as the vibrant breeding colors exhibited by mature males. Unlike other amphidromous euryhaline congeners, *R. duospilus* is a freshwater-dwelling species (Dudgeon and Corlett 2004; Ho and Dudgeon 2016). Such ecological habit results in highly fragmented habitats of this species, leading to genetic differentiation among populations across distinct environments (Wu et al. 2016). The most conspicuous manifestation of such differentiation lies in the morphological diversity observed between populations, particularly in body color polymorphism. This phenotypic variation poses challenges for taxonomy and precise management of population genetic resources of *R. duospilus*.

Currently, genetic studies on *R. duospilus* have been limited to local populations using restricted molecular markers (Wu et al. 2016). Although these studies have revealed interesting local population structure, significant knowledge gaps remain concerning the species' genome-wide genetic diversity and range-wide population structure. Moreover, the genetic basis underlying the observed morphological polymorphisms in *R. duospilus* has yet to be elucidated. Advances in high-throughput sequencing and assembly technologies have enabled genome studies to expand beyond evolution into areas such as gene function, ecology, and environmental adaptation. In light of these gaps, genome sequence with high-quality assembly and annotation of *R. duospilus* is critically needed. A well-characterized genome will serve as a foundational resource for elucidating the genetic mechanisms underlying population differentiation, morphological variation, and adaptive evolution through comparative genomics. Such data will significantly advance taxonomic classification, evolutionary research, and conservation genetics for this species.

## Materials and methods

### Sample collection, library preparation, and sequencing

**Fig. 1.**
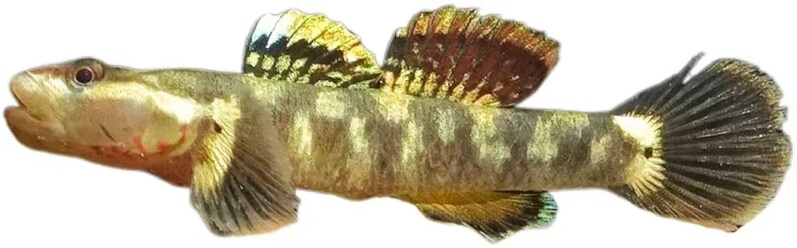
The specimen of *R. duospilus*.

A male *R. duospilus* (Fig. 1) with the standard length of 50 mm was collected from near its type locality in Shenzhen City, Guangdong Province, China, on 2024 September 1 (Table 1). From this specimen, we collected liver tissue for PacBio long-read sequencing and muscle tissue for Hi-C library construction and sequencing. In this process, the SMRTbell long-read sequencing library was constructed following the official protocol of the SMRTbell Template Prep Kit and sequenced on the PacBio Sequel II platform. Meanwhile, the Hi-C library was prepared using the restriction enzyme DpnII for digestion combined with formaldehyde cross-linking. The completed Hi-C library was sequenced on an Illumina sequencing platform by Novogene (Beijing, China). Furthermore, 8 additional tissue types were dissected for transcriptome analysis: eye, brain, gill, muscle, skin, liver, heart, and spleen. The RNA-seq libraries constructed from these tissue samples were sequenced on the Illumina platform as well. All library construction and sequencing procedures followed the manufacturer’s specified protocols using official reagent kits to ensure data quality and reproducibility.

**Table 1.** MIxS descriptors.

Item	Description
Investigation type	Vertebrate complete genome sequence
Project name	PRJNA1273456
Geographic location (latitude and longitude)	22.66 N, 113.97 E
Geographic location (country and/or sea, region)	China: Guangdong, Shenzhen
Collection date	2024 September 1
Broad-scale environmental context	Aquatic biome ENVO_00002030
Local environmental context	Freshwater river biome ENVO_01000253
Environmental medium	Freshwater ENVO_00002011
Sequencing method	PacBio HiFi sequencing Hi-C library
Assembly	hifiasm v0.16.0 Trinity v2.11.0

### Genome assembly of *R. duospilus*

Using the hifiasm v0.16.0 software (https://github.com/chhylp123/hifiasm) (Nishii et al. 2023), a preliminary genome assembly was performed with PacBio HiFi sequencing data. The assembled results were then indexed using BWA v0.7.17 (https://github.com/sghignone/bwa) (Li 2013) for subsequent analysis. Additionally, DpnII restriction enzyme cutting site information was generated for the genome, followed by executing an awk command to obtain length information for each sequence.

Subsequently, the Juicer v1.6 software (https://github.com/aidenlab/juicer.git) (Durand et al. 2016b) was employed to process Hi-C data, utilizing the preliminary genome assembly from hifiasm, the restriction enzyme cutting site information, and the sequence length information. This step generated an interaction matrix file for further assembly.

Using 3D-DNA v180922 (https://github.com/aidenlab/3d-dna.git) (Dudchenko et al. 2017), the genome sequence file and the interaction matrix produced by Juicer were combined to scaffold the genome sequences onto chromosomes, achieving chromosome-level genome assembly (parameter: *r* = 5). Juicebox v1.11.08 (https://github.com/theaidenlab/juicebox/wiki) (Durand et al. 2016a) was then used to manually correct scaffolding errors, thereby improving assembly accuracy. Finally, a Python script (https://github.com/luliangBio/3d-dna_tools) was called to integrate the results from Juicebox and hifiasm, generating the final chromosomal genome sequence.

The quality and completeness of the genome were assessed using Benchmarking Universal Single-Copy Orthologs (BUSCO) v5.5.0 (https://github.com/robsyme/busco.git) (Simão et al. 2015). A database named actinopterygii_odb10 was used for BUSCO analysis. The BUSCO database serves as a standardized, comprehensive, and reliable tool for effectively evaluating genome assembly completeness and annotation accuracy.

### Transcriptome assembly

Using the assembled genome of *R. duospilus* as the reference genome, BWA v0.7.17 (Li 2013) was employed to construct an index for the genome. Subsequently, the same software was used to align the transcriptome data to the reference genome, generating files in sam format. These sam files were then converted into sorted binary files in .bam format using SAMTools v0.1.19 (https://www.htslib.org/workflow) (Li et al. 2009) to facilitate downstream analysis. For the transcriptome assembly step, the Trinity v2.11.0 (https://github.com/trinityrnaseq/trinityrnaseq.git) software (Haas et al. 2013) was used to perform reference-based transcriptome assembly based on the bam files for sequences from 8 different tissues. The assembled sequences from these 8 tissues were then merged, and cd-hit (Li and Godzik 2006) was applied to remove redundancy from the merged Trinity assembly results. This process yielded a comprehensive transcriptome assembly, which was subsequently used for genome annotation.

### Gene prediction and annotation

Using RepeatMasker v4.1.0 (https://www.repeatmasker.org/), the genome was soft-masked by de novo repeat sequence database construction. Subsequently, protein databases of 6 closely related species were downloaded from the National Center for Biotechnology Information (NCBI), including *Periophthalmus magnuspinnatus* (GCA_009829125), *Boleophthalmus pectinirostris* (GCA_026225935), *Mugilogobius chulae* (GCA_046056395), *Pomatoschistus minutus* (GCA_009829125), *Eucyclogobius newberryi* (GCA_026437365), and *Danio rerio* (GCA_049306965). The protein sequences of these species were merged and deduplicated. The BRAKER3 pipeline (https://github.com/Gaius-Augustus/BRAKER.git) (Gabriel et al. 2024) was then employed to predict and annotate the genome of *R. duospilus*, integrating the soft-masked genome, the deduplicated protein database of related species, and the assembled transcriptome data. Finally, InterProScan (https://interproscan-docs.readthedocs.io/en/v5/) was used to analyze and summarize the annotation results. InterProScan integrates multiple databases. It is a powerful biological sequence analysis tool that consolidates various protein signature databases and predictive tools, such as GO, PANTHER, SMART, SUPERFAMILY, Gene3D, Pfam, ProSiteProfiles, and FunFam, among others.

### Mitochondrial genome assembly and annotation

The mitochondrial genome of *R. duospilus* was assembled using NOVOPlasty 4.3.1 (https://github.com/ndierckx/NOVOPlasty) with the COI gene of *Rhinogobius similis* as the seed. The coding sequence (CDS) were then annotated in Geneious (https://www.geneious.com/) using the complete mitochondrial genome of *R. similis* as a reference. Finally, annotations for rRNA, tRNA, and the D-loop control region were added by incorporating predictions from MITOS2 (https://gitlab.com/Bernt/MITOS/), yielding the complete mitochondrial genome annotation.

### Genome synteny analysis

The genome assembly and annotation files of *R. similis* were downloaded from the NCBI database (accession number: GCA_019453435.1). Chromosomal synteny analysis between *R. duospilus* and *R. similis* was then performed using MCScanX (https://github.com/wyp1125/MCScanX) under default parameters.

## Results and discussion

### Sequencing and genome assembly results

The PacBio HiFi sequencing achieved 100× coverage, which is sufficient for chromosome-level genome assembly. The HiFi sequencing data consisted of 51.5 Gb total base pairs from 3,145,229 reads, with maximum and mean read lengths of 59,675 bp and 16,374 bp, respectively, and a read length N50 of 16,291 bp. Additionally, the Hi-C data reached 100× coverage, indicating that the obtained Hi-C sequencing data were adequate for chromosome anchoring. The RNA-seq read length was 150 bp.

**Fig. 2.**
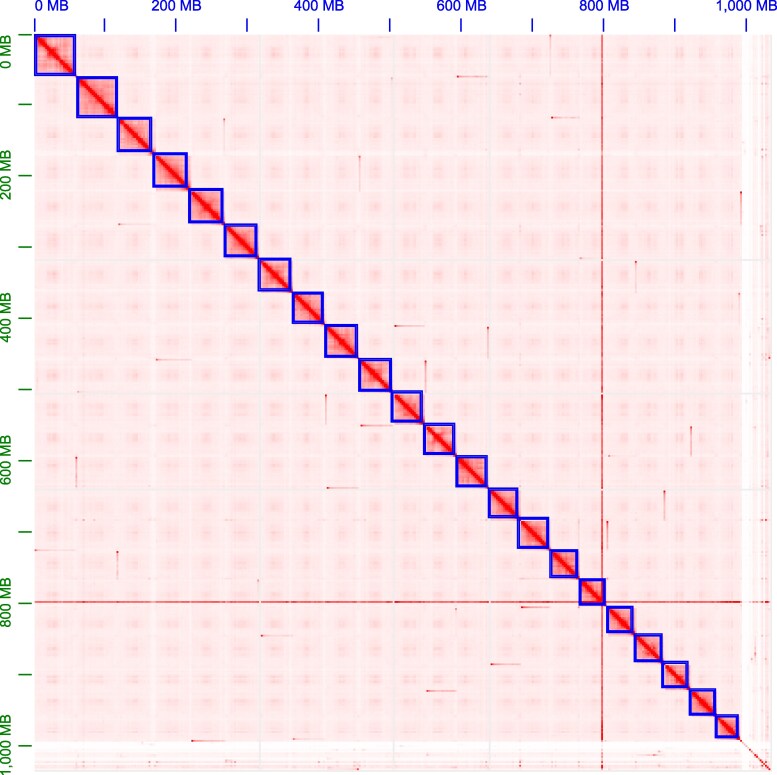
Hi-C map of *R. duospilus*.

**Fig. 3.**
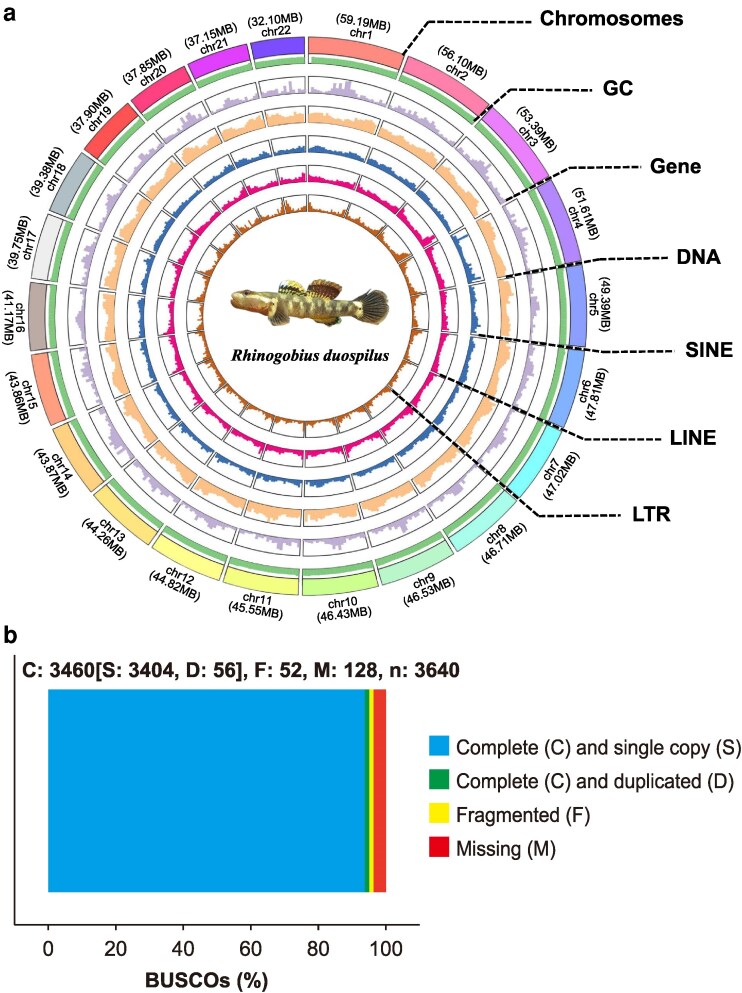
Chromosome-level assembly of *R. duospilus*. a) Circos plot of the *R. duospilus* genome, with visualization of chromosome sizes, GC content, gene density, DNA, SINE, LINE, and LTR in order from outside to inside. b) BUSCO evaluation results of the *R. duospilus* genome assembly.

The statistical results of the 2 haplotypes obtained by hifiasm assembly are presented in Table 2. The final assembled genome size was 1,031.61 Mb, with approximately 991.84 Mb (96.14%) of the sequences anchored to 22 chromosomes. The Hi-C heatmap is shown in Fig. 2. The undetermined sequences (N) accounted for only 0.02%, indicating a high-quality chromosome-level assembly of *R. duospilus* with superior accuracy. Furthermore, the high contig N50 (6.58 Mb) and scaffold N50 (45.55 Mb) values demonstrate excellent assembly quality. Notably, the scaffold N50 was significantly higher than the contig N50, indicating effective joining of multiple contigs and suggesting high sequence continuity in the assembly. The detailed assembly statistics are presented in Table 3, with the lengths of individual assembled chromosomes shown in Fig. 3a.

**Table 2.** Summary statistics of 2 haplotypes.

Attribute	Haplotype 1	Haplotype 2
Genome size	1.44 Gb	1.35 Gb
Scaffold N50	6.41 Mb	6.80 Mb
Number of scaffolds	2,491	1,311
Longest scaffold	33.81 Mb	40.82 Mb
GC content	40.60%	40.49%

**Table 3.** Summary statistics of the *R. duospilus* genome.

Attribute	Value
Size	1,031.61 Mb
Chromosome	22
Chromosome %	96.14%
Complete BUSCO (Actinopterygii odb10)	95%
Protein-coding genes	24,418
Annotated %	96.80%

The detailed BUSCO evaluation results of assembly are presented in Fig. 3b. The Complete BUSCOs accounted for 95% of the dataset, exceeding the 90% benchmark and slightly higher than the Complete BUSCO score (93.02%) reported for the congeneric species *R. similis* (Hu et al. 2022). These results demonstrate excellent genome completeness in *R. duospilus* assembly, confirming the high-quality genome assembly we achieved for *R. duospilus*.

### Gene prediction and annotation

A total of 24,418 genes were predicted, of which 23,660 (96.8%) were successfully annotated, indicating that the vast majority of gene sequences were matched to known databases. The assembled *R. duospilus* genome achieved consistently high annotation rates in 5 of the reference databases examined, including GO, PANTHER, SUPERFAMILY, Gene3D, and Pfam. The PANTHER database yielded the highest success rate at 89.9%, followed by Pfam at 85.2%, GO at 80.2%, Gene3D at 73.5%, and SUPERFAMILY at 68.9%.

### Mitochondrial genome assembly and annotation

The complete mitochondrial genome of *R. duospilus* was successfully assembled using NOVOPlasty 4.3.1. The genome is a circular DNA molecule with a total length of 16,485 bp. Annotation results revealed that it contains 13 protein-coding genes, 2 rRNAs, 22 tRNAs, and 1 D-loop control region. The gene composition and arrangement are consistent with the typical mitochondrial genome structure of vertebrates.

### Genome synteny analysis

**Fig. 4.**
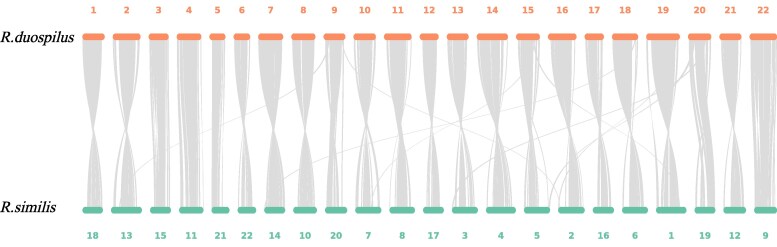
Synteny analysis between *R. duospilus* and *R. similis*.

The synteny analysis between *R. duospilus* and *R. similis* is shown in Fig. 4, revealing a high degree of synteny between 2 species. Chromosome fusion and fission events were observed, such as chromosome 9 of *R. duospilus* corresponding to chromosomes 13, 20, and 2 of *R. similis*. Similarly, chromosome 15 of *R. duospilus* corresponds to chromosomes 7, 5, and 1 of *R. similis*; chromosome 18 of *R. duospilus* corresponds to chromosomes 14 and 6 of *R. similis*; and chromosome 20 of *R. duospilus* corresponds to chromosomes 3, 5, 2, and 19 of *R. similis*.

### Conclusion

This study utilized PacBio HiFi sequencing technology and Hi-C data-assisted assembly to complete a chromosome-level genome assembly of the *R. duospilus*. Through genome assembly and gene prediction annotation, the research revealed the genome size and functional characteristics of genes. This study not only provides crucial genomic data for classification and evolutionary research of *R. duospilus* but also offers new insights into understanding the mechanisms of population differentiation and adaptation in this species.

## Data Availability

The data obtained in this study have been uploaded to the cited database. For additional information, please contact the authors. Raw sequencing data were deposited in the Sequence Read Archive (SRA) repository of NCBI, under the accession number SRR33889125, associated to the BioProject number PRJNA1273456 and BioSample number SAMN48946976. The raw transcriptome sequencing data are available under the accession numbers SRR33889116 to SRR33889123. The Hi-C data are available under the accession number SRR33905288. The assembled genome is available under the accession number JBPVZV000000000. The assembled transcriptome and assembled genome sequences have been deposited in the China National Center for Bioinformation (CNCB) under BioProject accession PRJCA043702. The assembled transcriptome is archived in the OMIX database with accession OMIX011202. The assembled genome and genome annotation file are deposited in the GWH database with BioSample SAMC5718041 and accession GWHGEXM00000000.1. The assembled complete mitochondrial genome has been submitted to the GenBase database under the accession number GB0006949 (https://ngdc.cncb.ac.cn/genbase/review/08785d6e9070). All data processing commands and pipelines followed the official manuals of the bioinformatics software (https://github.com/; https://www.htslib.org/workflow; https://www.repeatmasker.org/; https://interproscan-docs.readthedocs.io/en/v5/).
